# Predictors of segmental myocardial functional recovery in patients after an acute ST-Elevation myocardial infarction

**DOI:** 10.1016/j.ejrad.2019.01.010

**Published:** 2019-03

**Authors:** Kenneth Mangion, David Carrick, Guillaume Clerfond, Christopher Rush, Christie McComb, Keith G. Oldroyd, Mark C. Petrie, Hany Eteiba, Mitchell Lindsay, Margaret McEntegart, Stuart Hood, Stuart Watkins, Andrew Davie, Daniel A. Auger, Xiaodong Zhong, Frederick H. Epstein, Caroline E. Haig, Colin Berry

**Affiliations:** a British Heart Foundation Glasgow Cardiovascular Research Centre, University of Glasgow, UK; b West of Scotland Heart and Lung Centre, Golden Jubilee National Hospital, Clydebank, UK; c Clinical Physics, NHS Greater Glasgow and Clyde, Glasgow, UK; d Department of Biomedical Engineering, University of Virginia, Charlottesville, VA, USA; e MR R&D Collaborations, Siemens Healthcare, Los Angeles, CA, USA; f Robertson Centre for Biostatistics, University of Glasgow, UK

**Keywords:** AIC, akaike information ccriterion, DENSE, displacement encoding with stimulated echoes, LV, left ventricle, LVEF, left ventricular ejection fraction, MI, myocardial infarction, MRI, magnetic resonance imaging, STEMI, ST-segment elevation myocardial infarction, STEMI, Myocardial strain, Displacement encoding with stimulated echoes, DENSE

## Abstract

**Objective:**

We hypothesized that Displacement Encoding with Stimulated Echoes (DENSE) and feature-tracking derived circumferential strain would provide incremental prognostic value over the extent of infarction for recovery of segmental myocardial function.

**Methods:**

Two hundred and sixty-one patients (mean age 59 years, 73% male) underwent MRI 2 days post-ST elevation myocardial infarction (STEMI) and 241 (92%) underwent repeat imaging 6 months later.

The MRI protocol included cine, 2D-cine DENSE, T2 mapping and late enhancement.

Wall motion scoring was assessed by 2-blinded observers and adjudicated by a third. (WMS: 1=normal, 2=hypokinetic, 3=akinetic, 4=dyskinetic). WMS improvement was defined as a decrease in WMS ≥ 1, and normalization where WMS = 1 on follow-up. Segmental circumferential strain was derived utilizing DENSE and feature-tracking.

A generalized linear mixed model with random effect of subject was constructed and used to account for repeated sampling when investigating predictors of segmental myocardial improvement or normalization

**Results:**

At baseline and follow-up, 1416 segments had evaluable data for all parameters. Circumferential strain by DENSE (p < 0.001) and feature-tracking (p < 0.001), extent of oedema (p < 0.001), infarct size (p < 0.001), and microvascular obstruction (p < 0.001) were associates of both improvement and normalization of WMS. Circumferential strain provided incremental predictive value even after accounting for infarct size, extent of oedema and microvascular obstruction, for segmental improvement (DENSE: odds ratio, 95% confidence intervals: 1.08 per −1% peak strain, 1.05–1.12, p < 0.001, feature-tracking: odds ratio, 95% confidence intervals: 1.05 per −1% peak strain, 1.03–1.07, p < 0.001) and segmental normalization (DENSE: 1.08 per −1% peak strain, 1.04–1.12, p < 0.001, feature-tracking: 1.06 per −1% peak strain, 1.04–1.08, p < 0.001).

**Conclusions:**

Circumferential strain provides incremental prognostic value over segmental infarct size in patients post STEMI for predicting segmental improvement or normalization by wall-motion scoring.

## 1 Background

Early survival following an acute ST-segment elevation myocardial infarction (STEMI) has improved markedly in the past 3 decades in association with advances in pre-hospital emergency care and timely reperfusion therapy [1,2]. However, surviving patients have residual infarct pathology that predisposes to the subsequent development of left ventricular (LV) dysfunction and heart failure [3]. Recovery of myocardial pump function is associated with better clinical outcomes post-MI [4], and indices of LV function are a biomarker for the efficacy of novel therapies in clinical trials. In clinical practice, qualitative wall-motion scoring is generally used to assess LV systolic function post-MI [5,6].

The initial size of infarction is a determinant of prognosis [[7], [8], [9], [10], [11]]. In addition, parameters such as the extent of myocardial oedema [12], and the presence or absence of myocardial haemorrhage or microvascular obstruction [13] also have prognostic value for predicting recovery of function.

There is potential utility for strain to provide information over and above infarct characteristics to predict an improvement in wall motion scoring. Circumferential strain by tagging [8], but not by feature-tracking [11] provides incremental benefit over infarct size to predict an improvement in segmental wall motion scoring. A recent publication also describes the incremental utility of additional parameters (segmental extent of infarction, oedema, microvascular obstruction) for predicting recovery of segmental myocardial function [11].

Displacement encoding with stimulated echoes (DENSE) [14] is a non-contrast technique that directly reflects tissue displacement during the cardiac cycle which has been reported to have equal diagnostic utility as to myocardial tagging, which is regarded as the gold-standard of MRI strain methods. DENSE has equivalent or better accuracy and reproducibility of strain as compared to tagging [15,16], while providing simple and rapid strain analysis [[17], [18], [19]]. We aimed to build on the available evidence by performing an exploratory investigation comparing segmental oedema and infarct size, the presence or absence of microvascular obstruction, and segmental circumferential strain derived by feature-tracking and DENSE, to predict a reduction in segmental wall motion scoring and thus a recovery of myocardial function, and whether these parameters provided incremental benefit over segmental infarct size. Since strain values may differ between techniques, we used two independent methods to quantify strain.

## 2 Methods

### 2.1 Study population

We undertook a prospective single centre cohort study involving patients who underwent emergency invasive management for an acute STEMI. Patients with a contra-indication to cardiac magnetic resonance imaging (MRI), e.g. severe claustrophobia or a pacemaker were ineligible [20,21]. The study had ethics approval (reference 10-S0703-28) and was publicly registered (ClinicalTrials.gov identifier NCT02072850).

### 2.2 MRI acquisition

MRI was performed at 1.5 T (MAGNETOM Avanto, Siemens Healthcare, Erlangen, Germany) on a scanner located in a hospital Radiology Department, using an anterior phased-array body coil (12-element) and a posterior phased-array spine coil (24-element) 2 days and 6 months post-MI [6].

### 2.3 MRI protocol

The MRI protocol included cine (balanced steady-state free precession), mid-left ventricular 2D echo planar imaging (EPI) DENSE (work-in-progress sequence 611, Siemens Healthcare) [14,22], a T2-prepared balanced steady state free precession sequence (T2 map, Siemens Healthcare) [23,24], and late gadolinium enhancement (LGE) phase-sensitive inversion-recovery acquisitions [25] at baseline, and cine imaging at follow-up.

LV dimensions were assessed using b-SSFP cinematographic breath-hold sequences. The heart was imaged in multiple parallel short-axis planes 7-mm thick separated by 3-mm gaps. Typical imaging parameters were: repetition time 3.3 ms, echo time 1.2 ms, field of view 340 mm, flip angle 80, spatial resolution 180 × 256 mm, temporal resolution 46 ms, bandwidth 930 Hz/ pixel.

T2 maps were acquired in short-axis slices covering the whole ventricle, using a T2-prepared (T2P) balanced steady state free precession sequence (work-in-progress sequence 488, Siemens Healthcare, Erlangen, Germany) [23]. Typical imaging parameters were: bandwidth 947 Hz/pixel, flip angle 70, T2 preparations: 0, 24, and 55 ms, respectively, matrix 160× = 105 pixels, spatial resolution 2.6 × 2.1 × 8.0 mm, and slice thickness 8 mm.

Through-plane dephasing and 2-point complementary echo combination were used for artefact suppression during DENSE acquisition [26]. Fat suppression was carried out using a fast water excitation option. The readout and phase-encoding direction of displacement were acquired in a single breath-hold. DENSE imaging parameters were as follows: effective echo time 8 ms; repetition time 16.3 ms; flip angle 20°; slice thickness 8 mm; field of view 360 mm × 270 mm; matrix size 112 × 84; displacement encoding of 0.2 π/mm; EPI factor of 8 and views per segment of 16.

Late gadolinium enhancement images covering the entire LV were acquired 10–15 minutes after intravenous injection of 0.15 mmol/kg of gadoterate meglumine (Gd^2+^-DOTA, Dotarem, Guebert S.A.) using segmented phase-sensitive inversion recovery (PSIR) turbo fast low-angle shot in a contiguous short-axis LV stack and three orthogonal long-axis planes. Microvascular obstruction was defined as a dark zone on early delayed enhancement imaging 7 min post-contrast injection and within an area of late gadolinium enhancement. Typical imaging parameters were: matrix = 192 × 256, flip angle = 25°, TE = 3.36 ms, bandwidth = 130 Hz/pixel, echo spacing = 8.7 ms and trigger pulse = 2. The voxel size was 1.8 × 1.3 × 8 mm^3^. Inversion times were individually adjusted to optimize nulling of visually normal myocardium (typical values, 200–300 ms).

### 2.4 Image analysis

In order to reduce bias from variation in magnitudes of strain across the ventricle and inter-dependence of segmental values a single mid-ventricular slice-position was selected per participant. The mid-ventricular slice-position was identified prospectively by the scanning radiographer as the equidistant slice between the mitral valve plane and the left ventricular apex, for both baseline and follow-up scans. The superior right ventricular insertion point was utilized as a landmark for segmentation [27], and each image was segmented automatically into 6 segments of equal size (60°). Cine, T2, and late enhancement imaging at the same slice position were analysed. Mid-ventricular cine imaging from the 6-month MRI scan was reviewed visually to ensure that papillary muscle morphology and non-infarcted endocardial morphology were similar to ensure that the same ‘slice’ of myocardium was being analysed.

Data sets were anonymized to ensure operators were blinded to all other data. An independent biostatistician was responsible for data co-ordination and oversight.

#### 2.4.1 Wall motion scoring

Wall motion scoring was carried out by 2 cardiologists with >3 years of MRI experience blinded to all other data and adjudicated by a 3^rd^ expert observer with >10 years of MRI experience. Wall motion scoring (WMS) was defined as: 1 =normal, 2 =hypokinetic, 3 =akinetic, 4 =dyskinetic) [5].

Improvement in segmental function was defined as a decrease in WMS ≥ 1, and normalization of segmental function was defined as a WMS = 1 on follow-up when the baseline WMS had been ≥2.

#### 2.4.2 Tissue characterization

The segmental extent of scar revealed by late gadolinium enhancement imaging was assessed using the 5-standard deviation (SD) thresholding method (Qmass software Medis suite V2.1, Medis solutions, Leiden, the Netherlands) and expressed as a percentage of the myocardial segment [28]. Microvascular obstruction was defined as a hypo-intense core within a hyperenhanced region on late gadolinium enhancement imaging and expressed as present/absent.

The segmental extent of edema was assessed using the 2SD thresholding method [21] on T2 maps (QMass software) and expressed as a % of the myocardial segment.

#### 2.4.3 Circumferential strain

DENSE was analyzed using CIM_DENSE2D software (University of Auckland, New Zealand) as previously described [15,29] and expressed as percentage per segment. Greater magnitudes of circumferential shortening are reflected by a more negative value. Diogenes MRI feature-tracking software (TomTec Imaging Systems, Germany) was used to quantify regional strain from mid-LV short axis images spatially co-registered to the DENSE images. The same operator (K.M.) derived strain following a standard protocol taught by the software manufacturer [30].

### 2.5 Quality assessment: intra- and inter-observer variability

Two observers re-analysed 10 short axis scans (n = 60 segments) in random order separated by a 2-week interval. The following parameters were assessed: myocardial oedema (% LV mass), late gadolinium enhancement (% LV mass), microvascular obstruction (present/absent), and circumferential strain (%).

### 2.6 Statistical analysis

Normality was tested using the Kolmogorov-Smirnov test. Continuous variables were expressed as mean ± standard deviation (SD) or median (Q1, Q3) depending on distribution. Skewed distributions were analysed utilizing Mann-Whitney tests. A p-value of <0.05 was considered statistically significant. As the 6 segments per mid-left ventricular slice are inter-related, a generalized linear mixed model with random effect of subject was constructed and used to account for repeated sampling when investigating predictors of segmental myocardial improvement or normalization.

Akaike Information Criterion (AIC) [31] was used to assess relative model quality- the smaller the value the more robust the model’s predictive accuracy. Diagnostic cut-off values were identified from the ‘optimal-cut-points’ package [32] where specificity and sensitivity intersected. Statistical analysis was performed using R V.2.15 or higher (http://www.r-project.org).

## 3 Results

### 3.1 Characteristics of the study participants

**Fig. 1:**
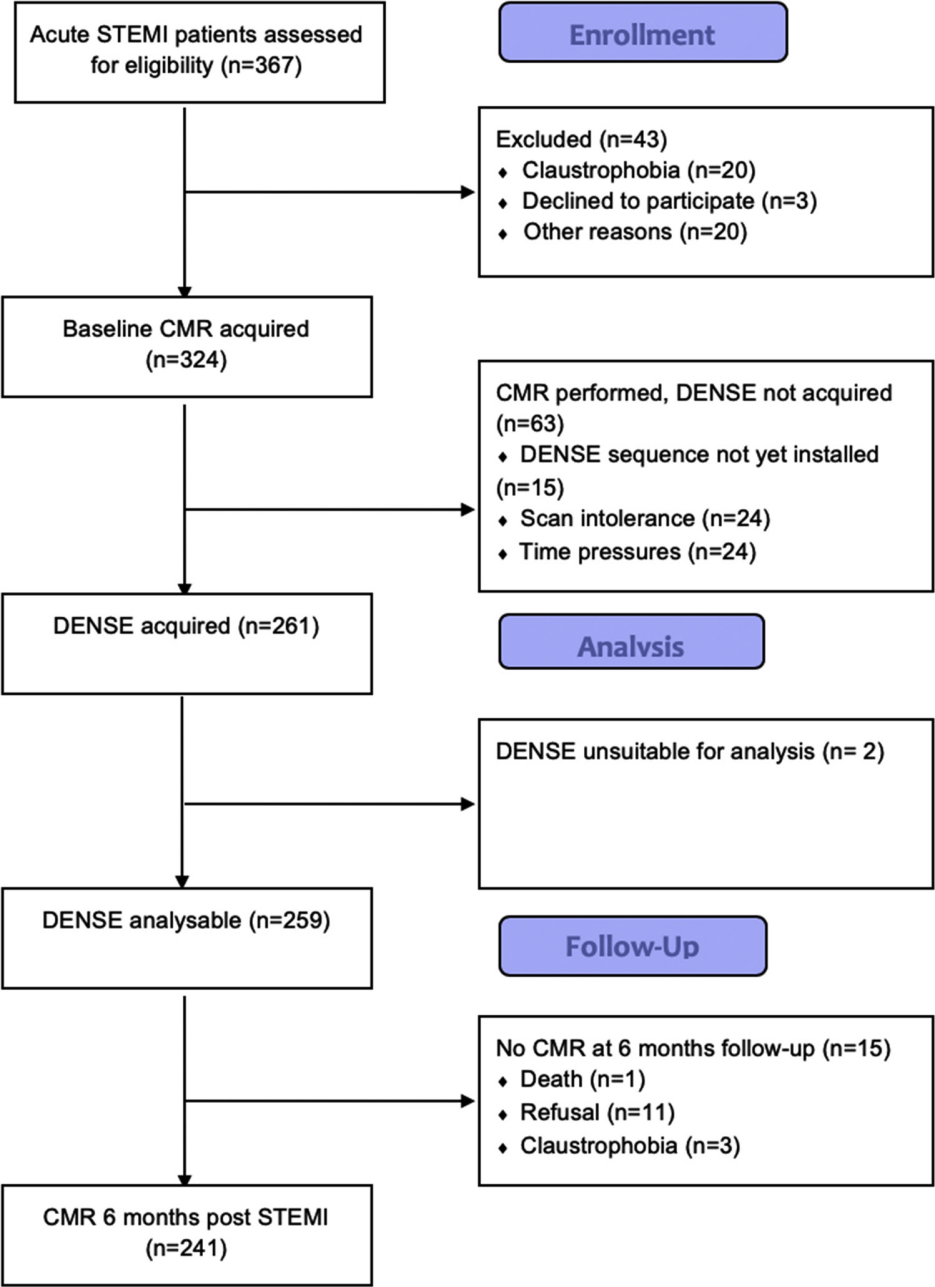
Study flow diagram.

**Fig. 2:**
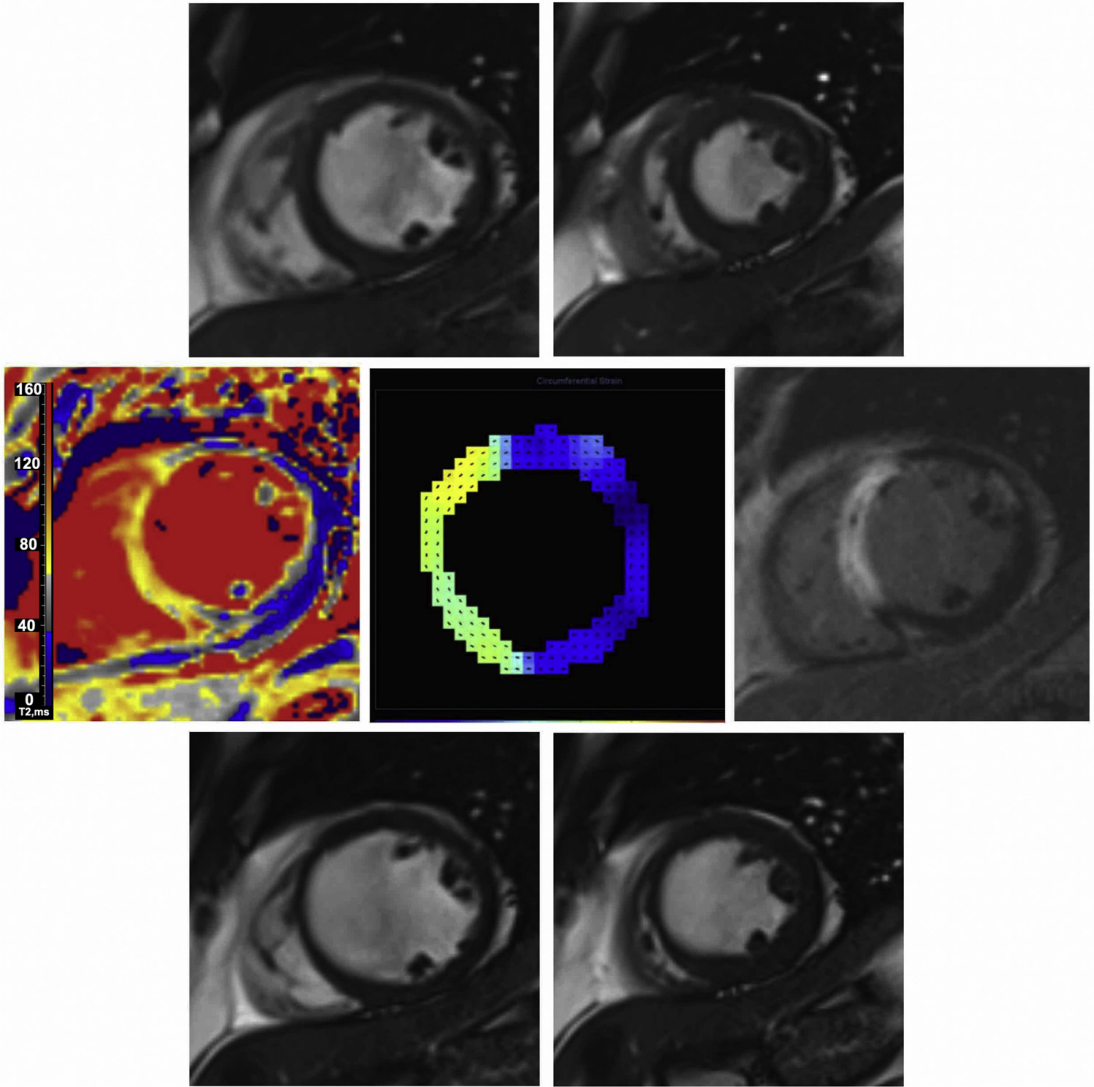
Infarct pathology and its prediction of segmental myocardial function. A 58 year old male presented with an anterior ST-elevation myocardial infarction, and had angioplasty to his left anterior descending coronary artery. He underwent an MRI scan on day 3 of his admission, and at 6 months follow-up. All the images are co-located, short-axis, mid-left ventricular images. Top row, shows cine imaging in diastole and systole. The anterior, antero-septal and infero-septal segments were scored as being akinetic by 2 independent observers. Middle row, left to right, demonstrates a T2 parametric map with a higher values (grey-yellow) signifying oedema in the region of the infarct. The middle figure is a DENSE peak end-systolic strain map, which illustrates the remote zone in blue and the infarcted region as yellow and green. The next image is late-gadolinium enhancement image, depicting a hyperintense infarcted region with some microvascular obstruction. At 6-month follow-up, (bottom row), the antero-septal region is still akinetic and thinned. The strain in the akinetic regions was under the cut-off (−8.17%) identified for segmental improvement. Strain map generated by Dr D.A. Auger through post-processing.

Two hundred and sixty one invasively managed patients with acute STEMI underwent cardiac MRI at 1.5 T 2.2 ± 1.9 days after hospital admission. Two patients (1%) had DENSE sequences of un-interpretable quality. Two hundred and forty-one (92%) patients attended for a follow-up scan (Fig. 1, Table 1) and represent the final study population. Fig. 2 depicts a case example.

**Table 1:** Demographics of the study population.

Characteristics^a^	Patients (n = 241)
Age, years	57.9 ± 11.1
Male sex, n (%)	184 (76)
BMI, (kg/m^2^)	28.5 ± 4.4
Hypertension, n (%)	75 (31)
Current smoking, n (%)	144 (60)
Hypercholesterolemia, n (%)	65 (27)
Diabetes mellitus^b^, n (%)	27 (11)
Previous angina, n (%)	27 (11)
Previous myocardial infarction, n (%)	13 (5)
Previous PCI, n (%)	12 (5)
*Presenting characteristics*	
Heart rate, bpm	77 ± 17
Systolic blood pressure, mmHg	136 ± 25
Diastolic blood pressure, mmHg	80 ± 14
Time from symptom onset to reperfusion, min	247 ± 201
*Killip class*^b^*, n (%)*	
I	173 (72)
II	53 (22)
III or IV	15 (6)
*ECG*	
ST segment elevation resolution post PCI, n (%)	
Complete, ≥70 %	115 (48)
Incomplete, 30% to <70%	92 (38)
None, ≤30%	33 (14)
*Coronary angiography*	
Reperfusion strategy, n (%)	
Primary PCI	227 (94)
Rescue PCI (failed thrombolysis)	10 (4)
Successful thrombolysis	4 (2)
Number of diseased arteries, n (%)^c^	
1	136 (56)
2	72 (30)
3	33 (14)
Culprit artery, n (%)	
Left anterior descending	91(38)
Left circumflex	43 (18)
Right coronary	107 (44)
TIMI coronary flow grade pre-PCI, n (%)	
0/1	179 (74)
2	43 (18)
3	19 (8)
TIMI coronary flow grade post-PCI, n (%)	
0/1	4 (2)
2	6 (2)
3	231 (96)
Medical therapy on discharge, n(%)	
Aspirin	241 (100)
Clopidogrel	241 (100)
Beta blocker	236 (98)
ACE-Inhibitor or Angiotensin Receptor Blocker	237 (98)
Statin	241 (100)

BMI - body mass index, ECG - electrocardiogram; PCI - percutaneous coronary intervention, STEMI - ST-segment elevation myocardial infarction, TIMI - Thrombolysis In Myocardial Infarction.

^†^Diabetes mellitus was defined as a history of diet-controlled or treated diabetes.

a Values are mean SD, n (%).

b Killip classification of heart failure after acute myocardial infarction: class I no heart failure; class II pulmonary rales or crepitations, a third heart sound, and elevated jugular venous pressure; class III acute pulmonary oedema; and class IV cardiogenic shock.

c Multivessel coronary artery disease was defined according to the number of stenoses > 50% of the reference vessel diameter as reported by the attending cardiologist.

### 3.2 Segments available for analysis and quality assessment

Overall, 1416 myocardial segments had complete analysis of circumferential strain, percentage extent of oedema, percentage scar, and presence/ absence of microvascular obstruction (all baseline), and LV wall motion scores at baseline & follow-up.

Intra and inter-observer variability were assessed for all segmental parameters (Supplementary Table 1).

### 3.3 Regional myocardial function

At baseline, on wall-motion scoring, 470 (33%) segments had wall-motion dysfunction [dyskinesia: 199 (14%), akinesia: 243 (17%), aneurysmal: 28 (2%)]

At 6-month follow-up, 341 (73%) of dysfunctional segments improved, of which 267 (78%) normalized and 74 (22%) experienced a reduction in their wall-motion score.

### 3.4 Relationship of strain and infarct pathology with wall-motion scoring

#### 3.4.1 Strain and infarct pathology

Moderate correlation was observed between segmental circumferential strain with DENSE and infarct size (R = 0.45, p < 0.001), segmental extent of oedema and infarct size (R = 0.62, p < 0.001) and segmental strain and extent of oedema (R = 0.50, p < 0.001). Weak correlation was observed between segmental circumferential strain with feature-tracking and infarct size (R = 0.39, p < 0.001), and segmental strain and extent of oedema (R = 0.40, p < 0.001).

#### 3.4.2 Wall motion, strain and infarct pathology

Segmental circumferential strain with DENSE had a moderate correlation with wall-motion score at baseline (R = 0.50, p < 0.001) and a weak correlation with wall-motion score at follow-up (R = 0.33, p < 0.001). Circumferential strain by feature-tracking had a moderate correlation with wall-motion score at baseline (R = 0.50, p < 0.001) and a weak correlation with wall-motion score at follow-up (R = 0.33, p < 0.001).

Infarct size had a moderate correlation with wall-motion scoring at baseline (R = 0.63, p < 0.001) and a weak correlation at follow-up (R = 0.48, p < 0.001). Extent of oedema had a moderate correlation with wall-motion score at baseline (R = 0.58, p < 0.001) and a weak correlation at follow-up (R = 0.39, p < 0.001).

#### 3.4.3 Wall motion scoring and infarct pathology

**Fig. 3:**
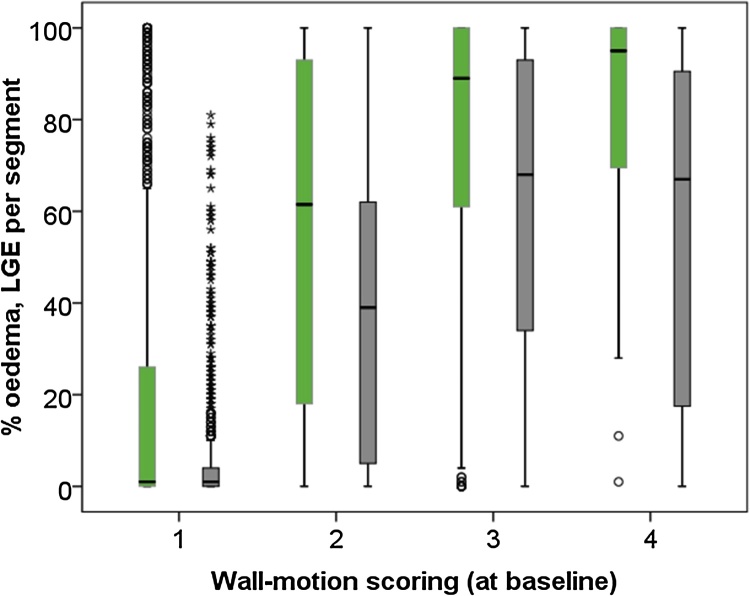
Oedema and infarct size divided according to wall-motion scoring at baseline. Segmental oedema (area-at-risk, green) and baseline infarct size (grey) increased as wall-motion scoring increased. A score of 4 was associated with a larger segmental infarct size and oedema.

Higher wall-motion scores were associated with a larger extent of oedema and infarct size (Fig. 3). Reductions in wall-motion scoring (i.e. an improvement in myocardial function at follow-up compared to baseline) occurred in 32 (89%) of dysfunctional segments with no evidence of infarction, 95 (89%) of segments with ≤25% infarct size, 65 (74%) of segments with 26–50% infarct size, 73 (76%) of segments with 51–75% infarct size, and 76 (55%) of segments with >75% infarct size.

Normalization in wall-motion scoring at follow-up compared to baseline occurred in 31 (86%) of dysfunctional segments with no infarction, 85 (79%) of segments with ≤25% infarct size, 52 (60%) of segments with 26–50% infarct size, 61 (48%) of segments with 51–75% infarct size, and 38 (27%) of segments with >75% infarct size.

Microvascular obstruction was present in 22 (15%) of segments with 26–50% infarct size, 61 (48%) of segments with 51–75% infarct size, and 38 (27%) of segments with >75% infarct size.

### 3.5 Univariate associates of segmental improvement by wall-motion scoring

Circumferential strain (DENSE, feature-tracking) (p < 0.001), extent of oedema (p < 0.001), infarct size (p < 0.001), and microvascular obstruction (p < 0.001) were all univariate associates of segmental improvement.

The strongest predictor, i.e. the one with the smallest AIC, was the segmental extent of oedema (Table 31). The area-under-the-curve (AUC) was used to derive cut-off values to predict segmental recovery (Table 2).

**Table 2:** Univariate predictors for myocardial segmental recovery by wall-motion scoring.

Segmental improvement							
Predictor	Odds Ratio	95% CI	P value	AIC	Cut-off value	Sensitivity	Specificity
Infarct size	1.04 per +1% infarct size	1.03–1.05	<0.001	1297	60 %	64%	64%
Extent of edema	1.04 per +1% extent of edema	1.03–1.05	<0.001	1221	88%	60%	59%
DENSE E_CC_	1.16 per −1% peak strain	1.13–1.20	<0.001	1336	−8.17%	56%	56%
Feature-tracking E_CC_	1.42 per −1% peak strain	1.06–1.91	<0.001	1367	−10.64	0.57	0.57
Microvascular obstruction	7.08 present / absent	4.45–11.25	<0.001	1447	NA		

Segmental normalization							
Infarct size	1.02 per +1% infarct size	1.02–1.03	<0.001	1262	55%	66%	65%
Extent of edema	1.03 per +1% extent of edema	1.02–1.03	<0.001	1194	85%	62%	63%
DENSE E_CC_	1.15 per −1% peak strain	1.11–1.18	<0.001	1245	−8.54%	58%	58%
Feature-tracking E_CC_	1.08 per −1% peak strain	1.06– 1.09	<0.001	1252	−10.64	0.57	0.57
Microvascular obstruction	3.33 present / absent	2.14–5.17	<0.001	1329	NA		

AIC- Akaike information criterion, CI- confidence intervals, E_CC_- circumferential strain.

### 3.6 Multivariate associates of segmental improvement by wall-motion scoring

Univariable associates of improvement in wall-motion score were added to infarct size to assess incremental utility in predicting segmental improvement (Table 3, Supplementary Table 2). Extent of oedema and circumferential strain were statistically significant multivariable associates in a model that included infarct size (p < 0.001), and their addition was accompanied by a decrease in the AIC of model, implying that these variables provided incremental predictive power over infarct size. Microvascular obstruction was not an incremental predictor over infarct size (p = 0.114).

**Table 3:** Multivariate predictors for myocardial segmental improvement by wall-motion scoring.

Segmental improvement				
Predictor	Odds Ratio	95% Confidence Intervals	P value	AIC
Infarct size, extent of edema, microvascular obstruction and DENSE				1179
Infarct size	1.02 per 1% infarct size	1.01–1.03	<0.001	
Extent of edema	1.02 per 1% extent of edema	1.02–1.03	<0.001	
Microvascular obstruction	0.50 present / absent	0.27K0.93	0.026	
Circumferential strain	1.08 per −1% peak strain	1.05–1.12	<0.001	

Infarct size, extent of edema, microvascular obstruction and feature-tracking				1159
Infarct size	1.01 per 1% infarct size	1.00–1.01	0.162	
Extent of edema	1.02 per 1% extent of edema	1.01–1.03	<0.001	
Microvascular obstruction	0.56 present/ absent	0.32–1.00	0.051	
Circumferential strain	1.05 per −1% peak strain	1.03–1.07	<0.001	

AIC- Akaike information criterion.

Circumferential strain from both DENSE and feature-tracking methods provided incremental predictive value for improvement even after accounting for infarct size, extent of oedema, and microvascular obstruction (p < 0.001).

### 3.7 Univariate associates of segmental normalization by wall-motion scoring

Circumferential strain by DENSE and feature-tracking (p < 0.001), extent of oedema (p < 0.001), infarct size (p < 0.001), and microvascular obstruction (p < 0.001) were associates of normalization of wall motion at 6 months. Extent of oedema was the strongest univariate predictor of this outcome based on the model with the smallest AIC.

### 3.8 Multivariate associates of segmental normalization by wall-motion scoring

After accounting for infarct size, circumferential strain and extent of oedema were significant associates of normalization of wall motion on a segmental basis (p < 0.001), improving the predictive ability of the model as reflected by the decrease in AIC. (Supplementary Table 3).

Circumferential strain by DENSE and feature-tracking provided incremental predictive power over infarct size, extent of oedema and microvascular obstruction, for normalization of segmental wall motion (Table 4).

**Table 4:** Multivariate predictors for myocardial segmental normalization wall-motion scoring.

Segmental normalization				
Predictor	Odds Ratio	95% Confidence Intervals	P value	AIC
Infarct size, extent of edema, microvascular obstruction and DENSE				1172
Infarct size	1.01 per 1% infarct size	1.00–1.01	0.154	
Extent of edema	1.02 per 1% extent of edema	1.01–1.03	<0.001	
Microvascular obstruction	0.55 present/ absent	0.30–1.00	0.049	
Circumferential strain	1.08 per −1% peak strain	1.04–1.12	<0.001	

Infarct size, extent of edema, microvascular obstruction and feature-tracking				1161
Infarct size	1.02 per 1% infarct size	1.01–1.02	<0.001	
Extent of edema	1.02 per 1% extent of edema	1.02–1.03	<0.001	
Microvascular obstruction	0.51 present/ absent	0.28–0.93	0.029	
Circumferential strain	1.06 per −1% peak strain	1.04–1.08	<0.001	

AIC- Akaike information criterion.

## 4 Discussion

Segmental circumferential strain revealed by two independent methods in patients two days post-STEMI provides incremental prognostic value over segmental infarct pathology for predicting improvement or normalization of wall motion at 6 months. To the best of our knowledge, other approaches for assessing LV function, including global indices, wall motion score, and other strain imaging techniques, have not been associated with incremental prognostic value over infarct size. Our results support the emerging role of strain imaging as a reference method for the assessment of regional myocardial contractility post-MI and for prognostication of regional LV function during longer-term follow-up. Whilst this does not equate with a prognostic advantage, an improvement in segmental myocardial function assessed with WMS is associated with a reduction in all-cause mortality and heart failure hospitalization [[33], [34], [35]].

Our study involving a comparatively large number of patients with acute STEMI extends the results of Wong et al. [8] who reported that circumferential strain with tagging provided incremental prognostic benefit in predicting segmental recovery in a group of 45 STEMI patients. Circumferential strain derived by feature-tracking [11], was not associated with incremental predictive utility over segmental infarct size to predict recovery or normalization of wall motion in 164 post-MI patients. The explanation for this discrepancy may relate to the strain methodologies. Feature-tracking estimates myocardial strain [36] by tracking border displacement and motion of columns of pixels rather than myocardial tissue, therefore theoretically reducing diagnostic accuracy and potentially, clinical utility [37,38]. Segmental strain assessment with feature-tracking is not as accurate or reproducible [37,[39], [40], [41]].

However, unlike Wong et al. [8] segmental circumferential strain with DENSE was a stronger univariate predictor for myocardial normalization, but a slightly weaker univariate predictor of myocardial improvement compared to infarct size.

Strain imaging with DENSE in patients after an acute STEMI has several potential benefits. We found that DENSE scans were well tolerated by patients, and with interpretable data gained in 95% of all subjects. Semi-automatic analysis of DENSE data is easier and faster when compared to tagging. This is the main limitation of the myocardial tagging technique.

### 4.1 Infarct

Infarct size is a determinant of prognosis, however, since infarct tissue is oedematous early post-MI, the initial extent of infarction by late gadolinium enhancement imaging may be over-estimated when compared to final infarct size 3–6 months later [[7], [8], [9], [10], [11]]. We found that segmental infarct size is a moderately strong predictor of potential for recovery by wall-motion scoring post-MI [11,42,43]. We used the segmental extent (absolute %) of late gadolinium enhancement as a measure of infarct size, rather than an ordinal score for the transmural extent of MI, which in our view is a more quantitative manner of describing infarct size on a segmental basis [11,42]. In our study, 56% of segments with ≥50% infarction experienced an improvement in function by wall-motion scoring, and 37% of segments with ≥50% infarction have a wall-motion score of 1 (i.e. normal) at follow-up. The cut-off for infarct size for segmental improvement was 60%. This confirms previous work reporting an overestimation in infarct size in the acute phase of myocardial infarction [7] and suggests that thresholds of infarct transmurality suggested for chronic infarction [44] may not be suitable in this patient group.

The absence of microvascular obstruction was a univariate predictor of myocardial recovery but was not associated with incremental prognostic benefit over and above segmental extent of infarction. This could be as microvascular obstruction is present mostly in transmural infarcts (88% was present in segments with >50% infarct size), thus offering minimal further information about the tissue. Kidambi et al. [13] reported that myocardial segments with microvascular obstruction were less likely to exhibit recovery, which could explain why microvascular obstruction was our weakest univariate predictor (i.e. as depicted by the largest AIC).

### 4.2 Oedema

The segmental extent of oedema was the strongest univariate predictor of myocardial recovery. These parameters were assessed using a T2 mapping technique, and all of the participants had analysable data. T2 mapping is a technique with emerging clinical utility. It is a more robust method than T2 short tau inversion recovery (T2-STIR) [25]. T2-STIR is potentially hampered by inadequate image quality, in part due to the low contrast-to-noise ratio between normal and abnormal myocardium [45].

## 5 Limitations

Our findings relate to imaging with MRI and may not be extrapolated to strain imaging using other methods. A limitation of this study is that only segmental strain data from mid-left ventricular slices was assessed per patient. We have performed a single centre study, and further research is warranted.

## 6 Conclusion

We found that the segmental extent of oedema assessed with T2 mapping is the strongest univariate predictor of segmental myocardial recovery or normalization by wall-motion scoring. Circumferential strain provides incremental prognostic benefit over segmental infarct size in predicting recovery.

## Ethics, consent and participation

The study had ethics approval (reference 10-S0703-28) and was publically registered (ClinicalTrials.gov identifier NCT02072850).

### Funding

This research was supported by the British Heart Foundation (BHF-PG/14/64/31043). Dr Mangion was supported by a Fellowship from the British Heart Foundation (FS/15/54/31639). Professor Berry was supported by a Senior Clinical Fellowship from the Scottish Funding Council.

## Authors’ contributions

KM, CH, CB made substantial contributions to conception and design.

DC, KGO, ML, MM, SH, MCP, SW, AD, CB made substantial contributions to acquisition of data;

KM, GC, CR, CM, DAA, FHE, CH, CB made substantial contributions to the analysis and interpretation of data;

KM, CH, CB drafted the article;

All authors were involved in revising it critically for important intellectual content;

All authors gave final approval of the version to be submitted and any revised version.

## Competing interests

Dr Xiaodong Zhong is an employee of Siemens Healthcare. The other authors report no competing interests. The University of Glasgow holds a research agreement with Siemens Healthcare, who provided the DENSE work-in-progress MRI sequence and data analysis software.
