# Supplementary material for: Predictors of segmental myocardial functional recovery in patients after an acute ST-Elevation myocardial infarction
Source: Eur J Radiol. 2019 Mar;112:121–9. doi: 10.1016/j.ejrad.2019.01.010 (PMC6390173; doi:10.1016/j.ejrad.2019.01.010)
Supplement: Supplementary file 1 [file mmc1.docx]

**Predictors of Segmental Myocardial Functional Recovery in Patients after an Acute ST-Elevation Myocardial Infarction**

## Online Supplement

### Supplementary Table 1. Segmental reproducibility analysis

| Variable | Mean bias ± SD | 95% Limits of agreement | ICC | Correlation | P value |
| --- | --- | --- | --- | --- | --- |
| DENSE- intra | 0.29 ± 1.84 | -3.39, 3.98 | 0.92 | 0.87 | <0.001 |
| DENSE- inter | 0.26 ± 2.30 | -4.36, 4.88 | 0.85 | 0.70 | <0.001 |
| Feature-tracking-intra | 2.51 ± 9.15 | -15.8, 20.8 | 0.87 | 0.79 | <0.001 |
| Feature-tracking-inter | 2.59 ± 10.2 | -16.3, 21.5 | 0.82 | 0.663 | <0.001 |
| Infarct size- intra | 1.25 ± 0.71 | -0.16, 2.66 | 0.98 | 0.96 | <0.001 |
| Infarct size- inter | -0.15 ± 7.09 | -14.33, 14.33 | 0.94 | 0.88 | <0.001 |
| Extent of oedema- intra | 2.00 ± 1.28 | -0.56, 4.56 | 0.97 | 0.96 | <0.001 |
| Extent of oedema- inter | 1.32 ± 7.66 | -14.00, 16.65 | 0.98 | 0.98 | <0.001 |
| MVO- intra* | - | - | 1.00 | 1.00 | <0.001 |
| MVO- inter* | - | - | 1.00 | 1.00 | <0.001 |

60 segments were used. ICC – intra-class correlation co-efficient, * Kappa statistic used as binary variable, DENSE- displacement encoding with stimulated echoes, DT- deformation-tracking, FT- feature-tracking, SD- standard deviation, ICC- intraclass correlation co-efficient

### Supplementary Table 2. Multivariate predictors for myocardial segmental improvement by wall-motion scoring.

| Segmental improvement | | | | |
| --- | --- | --- | --- | --- |
| Predictor | Odds Ratio | 95% Confidence Intervals | P value | AIC |
| Infarct size and microvascular obstruction | | | | 1297 |
| Infarct size | 1.04 per +1% infarct size | 1.03-1.05 | <0.001 |  |
| Microvascular obstruction | 0.62 present/ absent | 0.34-1.13 | 0.114 |  |
| Infarct size and extent of oedema | | | | 1201 |
| Infarct size | 1.02 per 1% infarct size | 1.01-1.02 | 0.09 |  |
| Extent of oedema | 1.03 per 1% extent of oedema | 1.02-1.04 | <0.001 |  |
| Infarct size, extent of oedema and microvascular obstruction | | | | 1200 |
| Infarct size | 1.02 per 1% infarct size | 1.01-1.03 | <0.001 |  |
| Extent of oedema | 1.03 per 1% extent of oedema | 1.02-1.03 | <0.001 |  |
| Microvascular obstruction | 0.55 present/ absent | 0.30-1.02 | 0.03 |  |

AIC - Akaike information criterion

### Supplementary Table 3. Multivariate predictors for myocardial segmental normalization wall-motion scoring.

| Segmental normalization | | | | |
| --- | --- | --- | --- | --- |
| Predictor | Odds Ratio | 95% Confidence Intervals | P value | AIC |
| Infarct size and microvascular obstruction | | | | 1263 |
| Infarct size | 1.03 per +1% infarct size | 1.03 1.02-1.03 | <0.001 |  |
| Microvascular obstruction | 0.66 present / absent | 0.37-1.18 | 0.158 |  |
| Infarct size and extent of oedema | | | | 1193 |
| Infarct size | 1.01 per 1% infarct size | 1.00-1.01 | 0.09 |  |
| Extent of oedema | 1.02 per 1% extent of oedema | 1.02-1.03 | <0.001 |  |
| Infarct size, extent of oedema and microvascular obstruction | | | | 1192 |
| Infarct size | 1.01 per 1% infarct size | 1.00-1.02 | 0.019 |  |
| Extent of oedema | 1.02 per 1% extent of oedema | 1.02-1.03 | <0.001 |  |
| Microvascular obstruction | 0.60 present/ absent | 0.33-1.08 | 0.088 |  |

AIC - Akaike information criterion
